# Combined Extra- and Intrapleural Hematoma After Blunt Chest Injury in an Anticoagulated Patient

**DOI:** 10.7759/cureus.5506

**Published:** 2019-08-28

**Authors:** Anupam Gupta, David Rubay, Daud Lodin, Robert Borrego, Lawrence Lottenberg

**Affiliations:** 1 Surgery, Charles E. Schmidt College of Medicine at Florida Atlantic University, Boca Raton, USA; 2 Surgery, Charles E. Schmidt College of Medicine, Florida Atlantic University, Boca Raton, USA; 3 Surgery, St. Mary's Medical Center, West Palm Beach, USA

**Keywords:** extrapleural, intrapleural, hematoma, warfarin

## Abstract

The objective of this study was to describe the atypical presentation of combined intrapleural and extrapleural hematomas in patients on anticoagulant therapy and explain the best workup and treatment for this pathology. This case report details the presentation, initial treatment, initial workup, and subsequent final treatment and workup of an elderly female patient that arrived at our trauma facility after suffering a blunt force trauma. The patient received anticoagulation therapy for her comorbidities prior to this incident. The outcome of interest was to better understand the best diagnostic and treatment modalities for treating combined intrapleural and extrapleural hematomas

## Introduction

Hemothorax is a life-threatening condition that can result after trauma. It can lead to severe respiratory compromise and circulatory failure. Blunt thoracic trauma comprises 25% of all trauma deaths worldwide [1]. Hemothorax results in a 30% (1500 to 2000 ml) total blood loss, typically presenting with hemorrhagic shock. Drainage and surgical intervention are paramount to stabilizing these patients [2]. Delayed treatment can lead to life-threatening complications, including severe infection, respiratory failure, and death [2]. In South Florida, our senior population is particularly prone to these complications as they are frequently treated with anticoagulants and antiplatelet agents for cardiac and vascular conditions. Patients on blood thinners can present with atypical sites of bleeding that require a careful radiographic evaluation and tailored treatment [3].

## Case presentation

**Figure 1:**
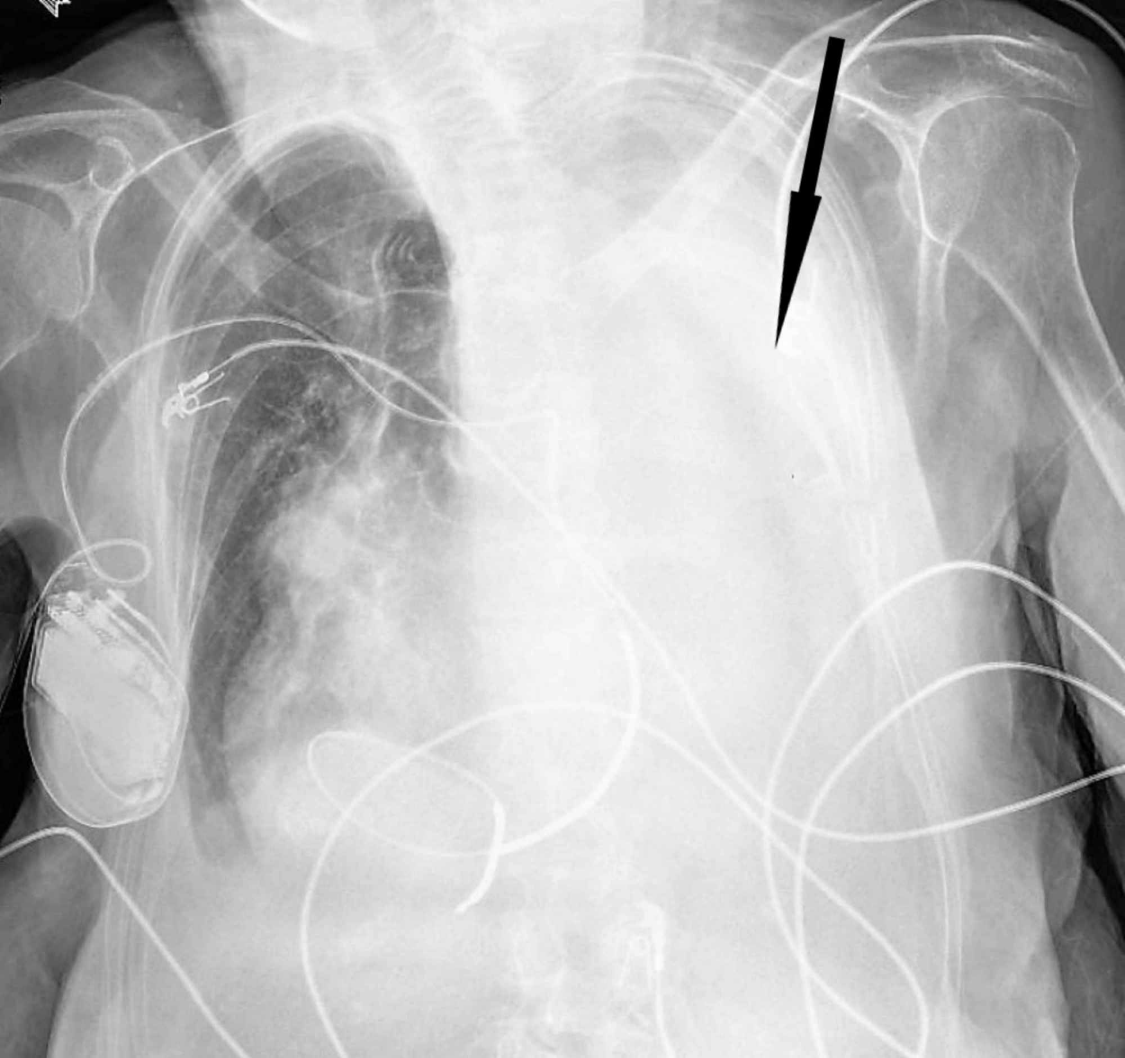
Chest X-ray on arrival

**Figure 2:**
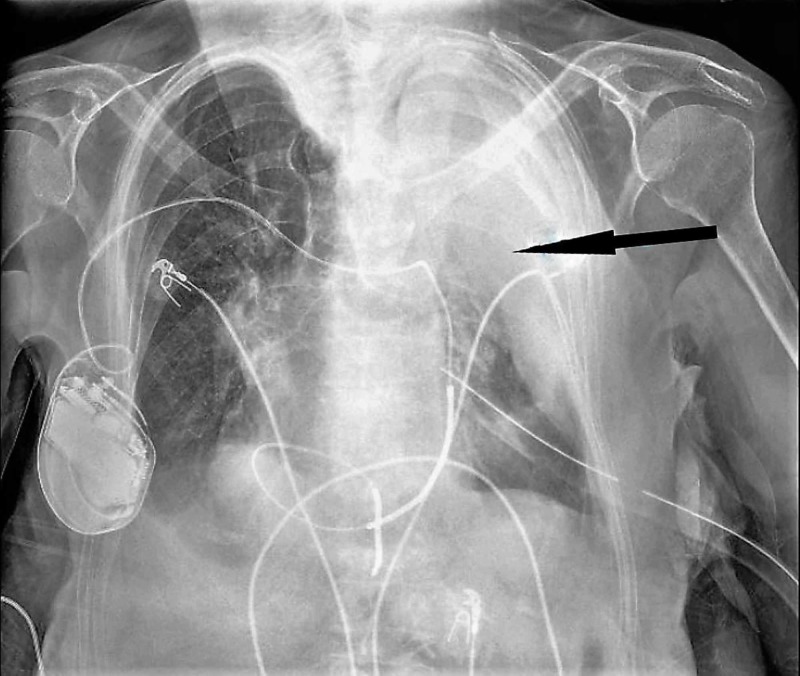
Chest X-ray after the placement of the chest tube

An 88-year-old Caucasian female presented to our hospital due to a left-sided hemothorax. The past medical history was significant for atrial fibrillation, and she was on a regiment of warfarin with an international normalized ratio (INR) goal greater than two for embolic prophylaxis. She sustained a mechanical fall from standing with no apparent neurologic consequences. Advanced trauma life support protocol was initiated on arrival (Figure 1). Her airway and breathing were maintained spontaneously. However, she arrived hypotensive with a blood pressure of 80 over 65. The remainder of her primary survey was negative. A portable X-ray in the trauma bay confirmed the presence of a left hemothorax. A single transfusion of packed red blood cells (PRBC) was able to stabilize her systolic blood pressure. A 36-French chest tube was placed with an immediate return of 800 mL of blood (Figure 2).

A follow-up chest X-ray indicated only a partial evacuation of the previously seen hemothorax with a significant amount of undrained blood. Blood work on arrival and before transfusion showed a white blood cell count of 15,000 per milliliter, a hemoglobin of 7.4 grams per deciliter, a hematocrit of 23.2%, a platelet count of 78,000, prothrombin time of 15.6, INR of 1.5, and partial thromboplastin time of 35.7. A thromboelastogram (TEG) done on arrival showed decreased maximum amplitude to 15.4 (normal >55). She received an additional "four-pack" platelets, one dose of prothrombin complex concentrate (Kcentra), and one dose of vitamin K to reverse her coagulopathy.

**Figure 3:**
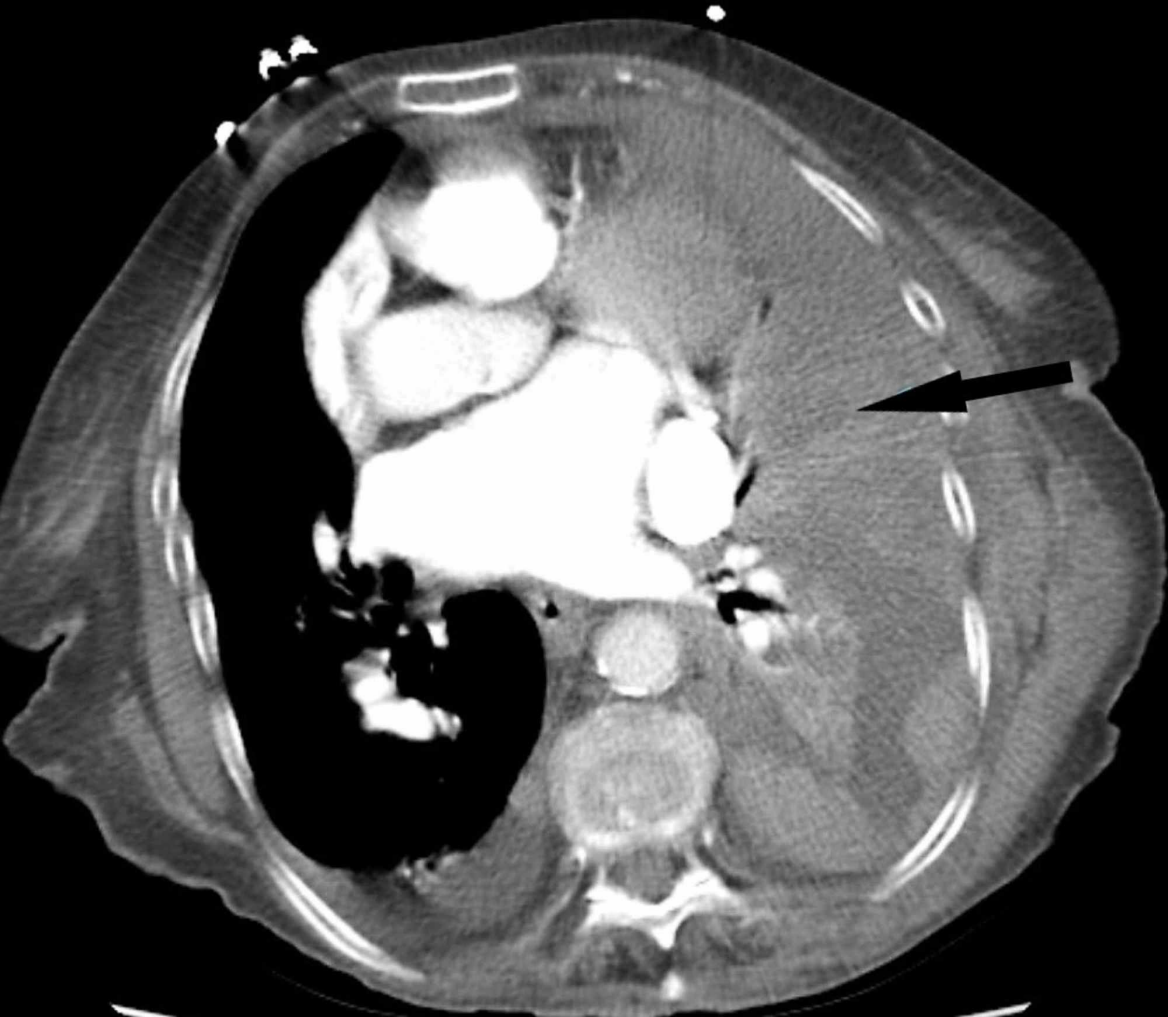
CT axial plane images section from prior to the placement of chest tube

**Figure 4:**
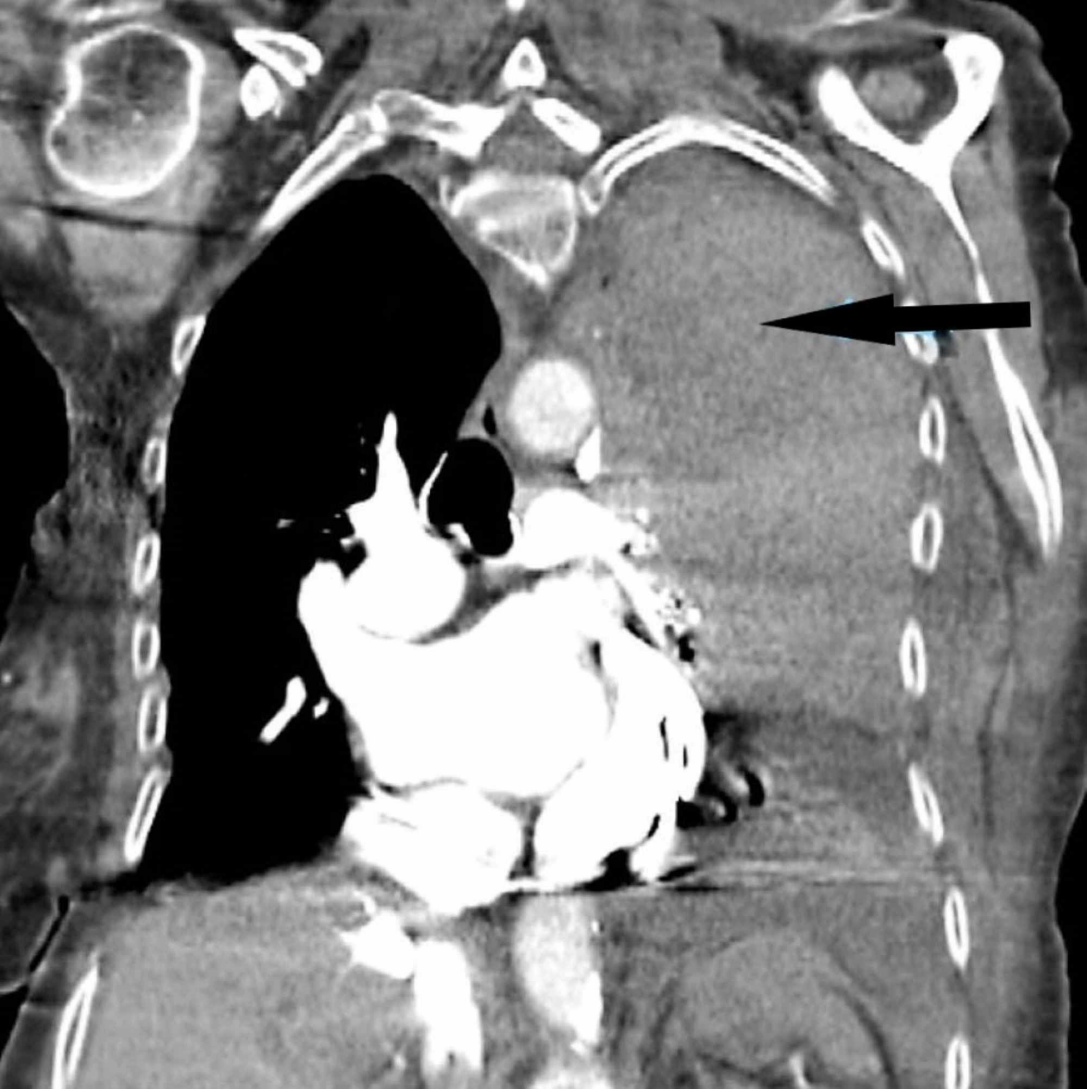
CT coronal plane image section post the placement of chest tube

After initial stabilization, computed tomography (CT) of the chest was performed, which revealed a combined intrapleural and extrapleural hematoma (Figures 3-4). The chest tube had successfully drained the intrapleural hematoma, but the extrapleural hematoma persisted. The patient was admitted to the surgical intensive care unit (SICU), and on hospital day two, the patient underwent open thoracotomy for the evacuation of her hematoma. Approximately two liters of clots were manually removed, a chest tube was placed in the patient’s extrapleural space. And underwent rib plating of left ribs 6 and 7 using the MatrixRIB Fixation System.

Oxygenation showed marked improvement after the procedure with PaO­_2_/FiO_2_ ratio jumping from 130 to 275 on a postoperative day (POD) 1. Her thoracostomy tubes were removed on POD seven, and the patient was successfully discharged to rehabilitation on POD eight. At the time of discharge, the patient’s final chest X-ray showed a complete resolution of her hematoma. 

## Discussion

Combined intrapleural and extrapleural hematoma in the setting of anticoagulation is an unusual presentation. A literature search on Pubmed using the keywords “combined extrapleural and intrapleural hematoma” yielded no results. Using the keywords “extrapleural hematoma,” 78 publications were found. A review of these papers showed 10 case reports, discussing 11 cases of extrapleural hematoma secondary to blunt trauma. Of these, three cases involved patients on anticoagulation [4-6]. In his autopsy study, Ezaki et al. identified a hematoma wall composed of parietal pleura, sub-pleura, an extrapleural fat layer, and endothoracic fascia. The medial displacement of the fat layer was noted on CT [7].

Injury to blood vessels of the chest combined with coagulopathy allows the development of hematomas that expand into the extrapleural space [3]. In a few cases described in our literature review, it was difficult to identify the placement of this hematoma by simple x-ray imaging [8]. On CT, an exclusive extrapleural hematoma is identified as a “fat sign” as the displacement of thoracic soft-tissue bands medially to the ribs due to fluid collection in the extrapleural space [9]. In cases of combined extrapleural and intrapleural hematoma, it may be difficult to identify this sign due to its unusual appearance on imaging. Simultaneous extrapleural and intrapleural hematomas obscure and hide the subpleural “fat sign” [10]. This problem was also noted in the CT read for our case. Early identification is difficult but important as an emergent intervention is necessary to prevent respiratory failure and circulatory collapse. In an elderly patient, who may already suffer from debilitating conditions, even minor trauma in the presence of coagulopathy can manifest itself as a more serious condition.

The use of INR and TEG can aid in the early identification of patients with pharmacological coagulopathy and allow for the reversal in a timely fashion for the patient that may suffer from the effects of a combined intrapleural and extrapleural hematomas [11]. Additionally, imaging modalities such as CT can help identify unusual locations of bleeding and aid in the diagnosis and early treatment. While intrapleural hematomas are typically treated with tube thoracostomies and video-assisted thoracoscopy (VATS), extrapleural hematomas are not amenable to these interventions [12]. Case studies focused on the combined presentation show management by observation, angioembolisation, VATS, and open thoracotomy [13]. Due to the limited number of documented and published combined hematoma cases, there is no consensus on management. The decision on what treatment modality to utilize must be based on the patient’s condition and the practitioner’s clinical experience [13].

Most authors agree that the management of these conditions can be successful with observation and tube thoracostomy in nonexpanding small hematomas in stable patients. One case report noted greater outcomes after encouraging better drainage through the introduction of thrombolytics via thoracostomy tubes [13]. For smaller hematomas, VATS was not considered a good treatment option, as it becomes difficult to visualize the anatomical cavity created by this combined hematoma. However, with larger hematoma cavities, VATS has been shown to be a viable option [4,12-13]. If VATS fails to provide adequate drainage due to difficult anatomic location or the presence of an extremely large hematoma, an open procedure may provide more expedited and complete drainage [13]. Treatment can be done in a stepwise fashion, where the intrapleural hematoma can be drained with a wide bore chest tube before a decision can be made on the management of the extrapleural portion of the hematoma. Despite these treatment solutions and strategies, early diagnosis remains a challenge for combined intrapleural and extrapleural hematomas.

## Conclusions

Patients with combined extrapleural and intrapleural hematoma do not have a typical clinical presentation. In senior or more elderly patients requiring pharmacological anticoagulation, this complicated chest pathology is likely to occur. The management of this problem relies on the reversal of coagulopathy, early intervention, and the treatment of the underlying trauma.
